# Predictive factors of coronavirus disease (COVID-19) vaccination series completion: a one-year longitudinal web-based observational study in Japan

**DOI:** 10.3389/fpubh.2024.1348170

**Published:** 2024-02-29

**Authors:** Takaomi Kobayashi, Mikiko Tokiya, Akiko Matsumoto, Takashi Nakano, Yoshio Hirota, Megumi Hara

**Affiliations:** ^1^Department of Preventive Medicine, Faculty of Medicine, Saga University Nabeshima, Saga, Japan; ^2^Department of Social and Environmental Medicine, Faculty of Medicine, Saga University Nabeshima, Saga, Japan; ^3^Department of Pediatrics, Kawasaki Medical School, Kurashiki, Japan; ^4^Clinical Epidemiology Research Center, SOUSEIKAI Medical Group (Medical Co. LTA), Fukuoka, Japan

**Keywords:** COVID-19, vaccination, completion, predictor, hesitant, social norms

## Abstract

**Introduction:**

Addresing vaccine hesitancy is considered an important goal in management of the COVID-19 pandemic. We sought to understand what factors influenced people, especially those initially hesitant, to receive two or more vaccine doses within a year of the vaccine’s release.

**Methods:**

We conducted longitudinal Web-based observational studies of 3,870 individuals. The surveys were conducted at four different time points: January 2021, June 2021, September 2021, and December 2021. In the baseline survey (January 2021), we assessed vaccination intention (i.e., “strongly agree” or “agree” [acceptance], “neutral” [not sure], and “disagree” or “strongly disagree” [hesitance]), and assumptions about coronavirus disease (COVID-19), COVID-19 vaccine, COVID-19-related health preventive behavior, and COVID-19 vaccine reliability. In subsequent surveys (December 2021), we assessed vaccination completion (i.e., ≥2 vaccinations). To investigate the relationship between predictors of COVID-19 vaccination completion, a multivariable logistic regression model was applied. Adjusted odds ratios (AORs) and 95% confidence intervals (CIs) were calculated while adjusting for gender, age, marital status, presence of children, household income category, and presence of diseases under treatment. In a stratified analysis, predictors were determined based on vaccination intention.

**Results:**

Approximately 96, 87, and 72% of those who demonstrated acceptance, were not sure, or hesitated had been vaccinated after 1 year, respectively. Overall, significant factors associated with COVID-19 vaccine compliance included the influence of others close to the index participant (social norms) (AOR, 1.80; 95% CI, 1.56–2.08; *p* < 0.001), vaccine confidence (AOR, 1.39; 95% CI, 1.18–1.64; *p* < 0.001) and structural constraints (no time, inconvenient location of medical institutions, and other related factors) (AOR, 0.80; 95% CI, 0.70–0.91; *p* = 0.001). In the group of individuals classified as hesitant, significant factors associated with COVID-19 vaccine compliance included social norms (AOR, 2.43; 95% CI, 1.83–3.22; *p* < 0.001), confidence (AOR, 1.44; 95% CI, 1.10–1.88; *p* = 0.008), and knowledge (AOR, 0.69; 95% CI, 0.53–0.88; *p* = 0.003).

**Discussion:**

We found that dissemination of accurate information about vaccines and a reduction in structural barriers to the extent possible enhanced vaccination rates. Once the need for vaccination becomes widespread, it becomes a social norm, and further improvements in these rates can then be anticipated. Our findings may help enhance vaccine uptake in the future.

## 1 Introduction

Controlling the new coronavirus disease (COVID-19) pandemic is an urgent issue that has necessitated the development and deployment of a vaccine. An effective vaccine for COVID-19 was developed within a year after the WHO declared it a pandemic (1–3), and has been administered worldwide (4). Shortly after its introduction, real-world effectiveness was reported, including its ability to prevent infection (5), onset (6), and disease severity (7) with two doses of vaccine (8, 9). In addition, there have been reports of the vaccine’s effectiveness in preventing post-infection sequelae (e.g., taste or smell alterations, post-exertional malaise, concentration difficulties, dyspnea, memory problems, fatigue, heart palpitations, vertigo or dizziness, hair loss, sleep disturbances, chest pain, swallowing difficulties) and cardiovascular diseases (e.g., ischemic and non-ischemic heart disease, dysrhythmias and others) (10–12), suggesting that the impact of the vaccines on the population may be even more significant over time. A modeling study assessing the effects of the first year of COVID-19 vaccination across 185 countries and territories estimated that the vaccine’s introduction saved approximately 19.8 million lives and led to a reduction of COVID-19-related deaths by approximately 63% over one year, and that regions with low vaccination rates tended to experience higher rates of COVID-19 (4). For a vaccine to be fully effective in a population, vaccination rates need to be sufficiently high (13).

In January 2019, the World Health Organization (WHO) included “vaccine hesitancy” in its list of the “ten threats to global health in 2019” (14); vaccine hesitancy is defined as a delay or refusal to accept vaccination despite the availability of vaccination services (15, 16). It is influenced by a complex interplay of factors such as geographical region, time period, the specific vaccine in question, individual knowledge and beliefs, trust in vaccines and public health institutions, and the convenience of accessing vaccination services (17, 18). Notably, when the results of 290 vaccine confidence surveys conducted in 149 countries worldwide from 2015 to 2019 were compiled, Japan was reported to have the lowest confidence rate (19). In a January 2021 Web survey of the Japanese general population, 51.4% of respondents expressed vaccine hesitancy (i.e., they disagreed or strongly disagreed) or were not sure about receiving the COVID-19 vaccine (18). Nevertheless, once vaccination began in June 2021, the number of people getting vaccinated increased among those who were hesitant or unsure whether to get vaccinated in January 2021. Subsequently, we conducted monitoring of COVID-19 vaccine hesitancy in Japan across three phases: vaccine approval, introduction, and deployment (20); our findings revealed a temporary increase in vaccine hesitancy during the introduction phase, which subsequently decreased during the deployment phase. By October 2023, approximately 80% of the entire population and >90% of older adult individuals had received at least one dose according to the Vaccination Record System (21). Conducting a pre- and post-vaccination survey in Japan to understand why hesitant individuals eventually accepted the vaccine and what discouraged others from getting vaccinated can be crucial in promoting timely vaccine uptake during future pandemics.

Although the infection rate of COVID-19 increased with each wave of the epidemic (22), the epidemic was brought under control as vaccination progressed until the appearance of the Omicron strain in 2022 (23). Regarding vaccine safety, minor adverse effects (i.e., injection site discomfort and fatigue) were reported to be the most commonly observed for mRNA, non-replicating viral vector, inactivated, and protein subunit-based vaccines (24). Additionally, simultaneous vaccination with influenza vaccine was recommended, for which no major safety issues were reported (25). Here, with the goal of helping to improve vaccine uptake in future pandemics, we endeavored to understand what factors influenced people, especially those initially hesitant, to complete their two vaccine doses within a year of the vaccine’s release.

## 2 Materials and methods

### 2.1 Study design and subjects

We conducted a Web-based observational study consisting of four separate surveys in 2021 prior to and following first-dose vaccine authorization in Japan. We followed the Strengthening the Reporting of Observational Studies in Epidemiology (STROBE) guidelines (26).

**Figure 1:**
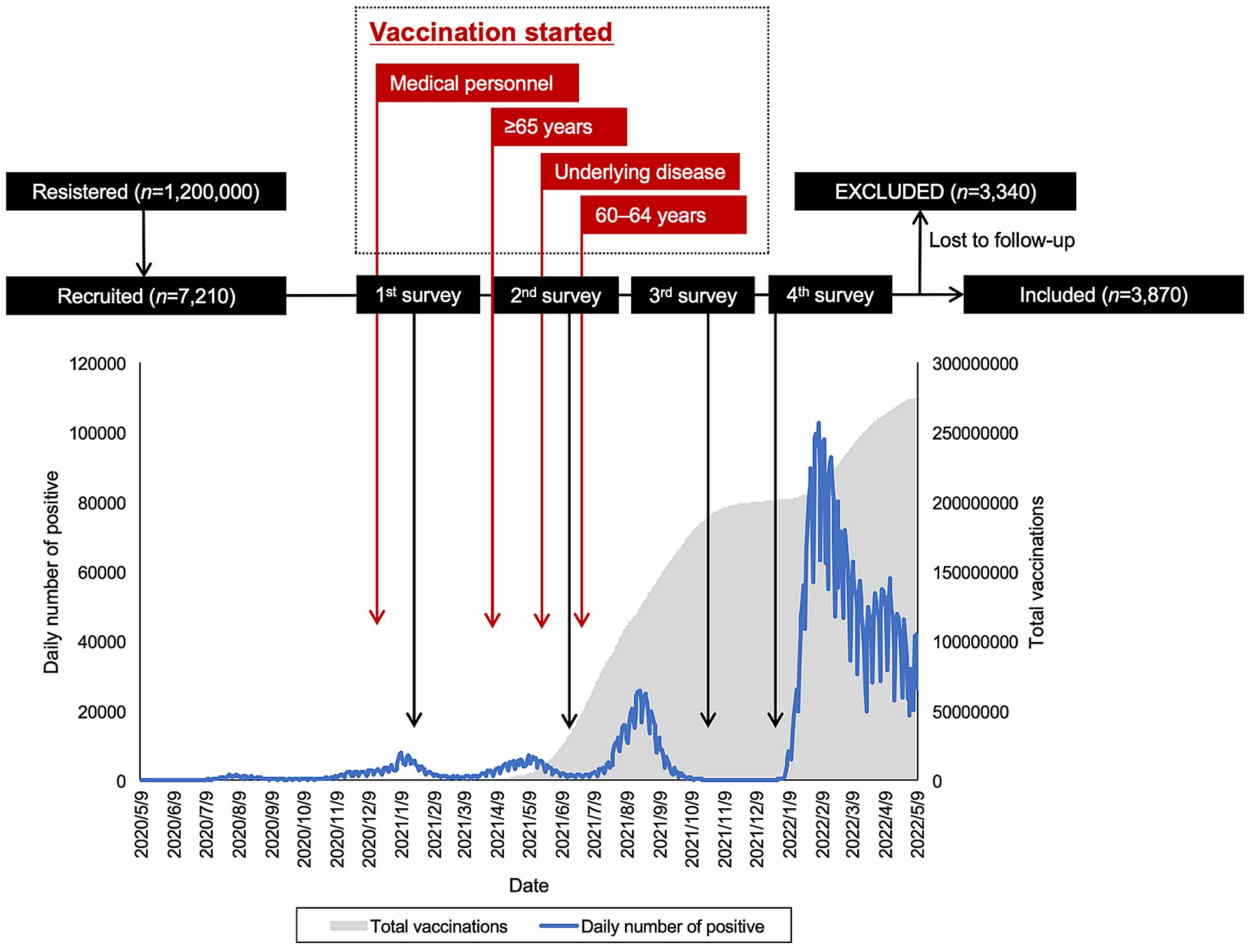
Flowchart of the study, including daily count of COVID-19-positive cases and total vaccinations.

The surveys were conducted at four different time points: January 2021, June 2021, September 2021, and December 2021. The baseline survey took place on January 19 and 20, 2021, preceding the approval of the COVID-19 vaccine in Japan in February 2021 (18, 25). The second survey occurred from June 23 to 24, coinciding with the period when vaccinations for medical workers were being completed, and vaccinations for older adult individuals were commencing (21). The third survey was conducted from September 27 to 29. These three survey periods coincided with the third, fourth, and fifth waves of COVID-19 in Japan, respectively. The fourth and final survey was carried out from December 20 to 22, just before the commencement of booster vaccination for the general adult population and before the approval of the COVID-19 vaccine for children aged 5 to 11 in Japan (27). The survey included participants of both genders aged 20 to 79 who were registered with an online research company, Macromill Co., Ltd., Tokyo. This company had a pool of 1.2 million registered users, and their gender, age, and regional distribution were adjusted to align with Japan’s population structure. A total of 7,210 individuals were recruited based on sample size calculations (18, 21, 28). From this group, 3,870 individuals who participated in all four surveys were included in the analysis for this study (Figure 1).

This study was approved by the Saga University Ethics Committee (approval number: R2-24) on November 30, 2020, and was conducted in accordance with the principles outlined in the Declaration of Helsinki.

### 2.2 Sociodemographic factors

Sociodemographic factors in this study included gender, age, area of residence (Hokkaido/Tohoku/Kanto/Chubu/Kinki/Chugoku/Shikoku/Kyushu), marital status, presence of children, household income category (<¥4 million/≥¥4 million), and educational background (high school/higher education). Additionally, body mass index (>25 kg/m^2^/≤25 kg/m^2^), diseases under treatment (Yes/No), and smoking status (Yes/No) were considered.

### 2.3 Number of vaccinations

Subsequent surveys in June 2021, September 2021, and December 2021 aimed to determine the number of COVID-19 vaccine doses received. Respondents could select from the options “None,” “One dose,” “I have been vaccinated twice,” and “I have been vaccinated three times”.

### 2.4 Relevant factors

Table 1 shows the results of a baseline survey (January 2021) for COVID-19 (8 items) (20), COVID-19 vaccine (7 items) (21), COVID-19-related health preventive behavior recommended by the Japanese Ministry of Health, Labor and Welfare (8 items), and COVID-19 vaccine reliability (14 items) (18, 26, 27). Respondents provided their answers using 5-point Likert scales, ranging from strongly disagree (1 point) to strongly agree (5 points).

**Table 1:** Relevant factors regarding COVID-19 assessed in this study in January 2021.

*Recognition of COVID-19 (8 items)*	
1. Knowledge	I know a lot about COVID-19.
2. Symptoms	All COVID-19 patients have symptoms.
3. Mild illness	Many people with COVID-19 have mild symptoms.
4. Severe illness	COVID-19 are more severe in people over 65 years old and those with chronic illnesses.
5. Easily infectious	COVID-19 is easily spread from person to person.
6. Worried about getting	I am worried about getting COVID-19.
7. May get	I may get COVID-19.
8. Repeated infections	Once you have COVID-19, you cannot get it again.
*Awareness of the COVID-19 vaccine (7 items)*	
1. Preventing severe illness	Preventing inoculated individuals from becoming seriously ill with COVID-19.
2. Prevention of infection	Prevention of COVID-19 in vaccinated persons.
3. Preventing relatives’ infection	Prevent family members and friends of the inoculated person from contracting COVID-19.
4. Prevention of spread	Prevent the spread of COVID-19 in the vaccinated person.
5. Adverse reactions	I am concerned about adverse reactions to the COVID-19 vaccine.
6. Fever or swelling	You may experience fever or swelling at the vaccination site after receiving the COVID-19 vaccine.
7. Social norms	I would like to be vaccinated if everyone else is vaccinated.
*Perceptions of COVID-19-related health preventive behavior (8 items)*	
1. Social distance	Avoiding crowded places and maintaining social distancing.
2. Handwashing	Practicing thorough handwashing for at least 20 s.
3. Hand sanitizer	Using hand sanitizer.
4. Wearing a mask	Consistently wearing a mask when interacting with others.
5. Indoor ventilating	Regularly ventilating and disinfecting indoor spaces.
6. Avoiding gatherings	Avoiding gatherings with more than five people.
7. Going out	Refraining from going out when feeling unwell.
8. Information	Staying informed about COVID-19 through regular updates.
*Perceptions of COVID-19 vaccine reliability (14 items)*	
1. Importance	Vaccines are important for my health.
2. Effectiveness	Vaccines are effective.
3. Herd immunity	My vaccination is important for the health of others in my community.
4. Risk	New vaccines carry more risks than older vaccines.
5. Anxiety	I am concerned about serious adverse effects of vaccines.
6. Trust	I do not need vaccines for diseases that are no longer common.
7. Confidence	Vaccines are safe.
8. Distrust	Serious adverse events may occur due to the vaccination.
9. Structural constraints	I have difficulty getting immunized (no time, inconvenient location of medical institutions, and other related factors).
10. Psychological constraints	We do not need to take voluntary vaccination.
11. Compliance	I do not want the vaccine if everyone around me is immunized.
12. Literacy	It is easy to obtain correct information on immunization.
13. Understanding necessity	It is easy to understand why immunization is needed.
14. Understanding vaccinations	I have been able to accurately understand the vaccinations I have received

COVID-19, coronavirus disease.

### 2.5 Vaccination intention

In the baseline survey (January 2021), vaccination intention was assessed as previously described (18, 21). Respondents were asked, “Would you definitely want to get vaccinated if a COVID-19 vaccine is approved?” They provided answers on a Likert scale, where 5 points indicated “strongly agree” (acceptance), 4 points were for “agree” (acceptance), 3 points represented “neutral” (not sure), 2 points corresponded to “disagree” (hesitance), and 1 point signified “strongly disagree” (hesitance).

### 2.6 Statistical analyses

Based on the baseline survey conducted in January 2021, all participants were grouped into five categories according to their vaccination intention, which was characterized as follows: strongly agree, agree, not sure, disagree, and strongly disagree. Among these five groups, sociodemographic factors were assessed; qualitative data were presented as numbers (percentages) and assessed using the chi-squared test, while quantitative data (with a non-normal distribution) were described as medians (ranges) and analyzed using the Kruskal-Wallis test.

For each of the surveys conducted in June 2021, September 2021, and December 2021, we investigated the percentage of individuals who had received ≥2 vaccinations based on their vaccination intention as recorded in the survey conducted before vaccine approval in January (i.e., strongly agree, agree, not sure, disagree, and strongly disagree). We performed this analysis using the chi-squared test.

To investigate the relationship between predictors of COVID-19 vaccination completion (i.e., ≥ 2 vaccinations as of December 2021), a multivariable logistic regression model using a backward stepwise algorithm was applied. The dependent variable was defined as the presence or absence of ≥2 doses of vaccine as of December 2021. The independent variables were defined from the Web-based surveys for COVID-19 (8 items), COVID-19 vaccine (7 items), COVID-19-related health preventive behavior (8 items), and COVID-19 vaccine reliability (14 items) as of the baseline survey (January 2021). Adjusted odds ratios (AORs) and 95% confidence intervals (CIs) were calculated while adjusted for gender, age, marital status, presence of children, household income category, and presence of diseases under treatment. In a stratified analysis, predictors were determined based on vaccination intention in January 2021, with categorization into three groups (i.e., hesitance, not sure, and acceptance).

The significance level (two-tailed *p*-value) was adjusted using Bonferroni correction as needed. SAS version 9.4 (SAS Institute Inc., Cary, NC, United States) was used for statistical analyses.

## 3 Results

### 3.1 Demographics based on vaccination intention pre-approval

A total of 3,870 individuals completed the longitudinal observational study. A total of 3,340 were lost to follow-up. Of the 3,870 individuals, 48.3% were female and median age was 53.0 years. Demographic information stratified by vaccination intention before vaccine approval is summarized in Table 2. Of 3,870 individuals, 538 (13.9%) strongly agreed, 1,360 (35.1%) agreed, 1,353 (35.0%) were neutral, 422 (10.9%) disagreed, and 197 (5.1%) strongly disagreed with vaccination. There were significant differences in gender (*p* < 0.001), age (*p* < 0.001), marital status (*p* < 0.001), presence of children (*p* < 0.001), household income (*p* = 0.001), and presence of diseases under treatment (*p* < 0.001).

**Table 2:** Demographics of individuals based on their vaccination intention prior to vaccine approval.

	Strongly agree (*n* = 538)	Agree (*n* = 1,360)	Neutral (*n* = 1,353)	Disagree (*n* = 422)	Strongly disagree (*n* = 197)	*p* value^a^
Female, *n* (%)	208 (38.7)	600 (44.1)	729 (53.9)	227 (53.8)	107 (54.3)	<0.001^b^
Age, years	55 (21–79)	55 (20–79)	50 (20–79)	51 (20–79)	48 (20–78)	<0.001^c^
Area						0.195^b^
Hokkaido, *n* (%)	26 (4.8)	61 (4.5)	66 (4.9)	20 (4.7)	11 (5.6)	
Tohoku, *n* (%)	36 (6.7)	81 (6.0)	57 (4.2)	15 (3.6)	7 (3.6)	
Kanto, *n* (%)	227 (42.2)	544 (40.0)	488 (36.1)	154 (36.5)	76 (38.6)	
Chubu, *n* (%)	72 (13.4)	225 (16.5)	236 (17.4)	78 (18.5)	26 (13.2)	
Kinki, *n* (%)	102 (19.0)	242 (17.8)	284 (21.0)	91 (21.6)	43 (21.8)	
Chugoku, *n* (%)	21 (3.9)	65 (4.8)	67 (5.0)	24 (5.7)	13 (6.6)	
Shikoku, *n* (%)	11 (2.0)	36 (2.7)	45 (3.3)	10 (2.4)	8 (4.1)	
Kyusyu, *n* (%)	43 (8.0)	106 (7.8)	110 (8.1)	30 (7.1)	13 (6.6)	
Married, *n* (%)	372 (69.1)	922 (67.8)	827 (61.1)	247 (58.5)	115 (58.4)	<0.001^b^
Children, *n* (%)	357 (66.4)	883 (64.9)	770 (56.9)	231 (54.7)	93 (47.2)	<0.001^b^
Household income						0.001^b^
<¥4 million, *n* (%)	137 (25.5)	349 (25.7)	377 (27.9)	121 (28.7)	67 (34.0)	
≥¥4 million, *n* (%)	304 (56.5)	744 (54.7)	655 (48.4)	208 (49.3)	83 (42.1)	
Unknown, *n* (%)	97 (18.0)	267 (19.6)	321 (23.7)	93 (22.0)	47 (23.9)	
Educational background						0.934^b^
High school	158 (29.4)	411 (30.2)	415 (30.7)	132 (31.3)	56 (28.4)	
Higher education	380 (70.6)	949 (69.8)	938 (69.3)	290 (68.7)	141 (71.6)	
Body mass index >25 (kg/m^2^)	125 (23.2)	287 (21.1)	276 (20.4)	68 (16.1)	37 (18.8)	0.087^b^
Diseases under treatment	260 (48.3)	576 (42.4)	451 (33.3)	150 (35.6)	58 (29.4)	<0.001^b^
Smoking, Yes	95 (17.7)	237 (17.4)	234 (17.3)	55 (13.0)	28 (14.2)	0.189^b^

a Significance level (*p*-value) was set at 0.05.

b Values are presented as number (percentage) and compared using the chi-squared test.

c Values are presented as median (range) and compared using the Kruskal-Wallis test.

Supplementary Table S1 displays the percentage of responses to each COVID-19-related questionnaire, stratified by vaccination intention before vaccine approval. All questionnaire items demonstrated significant differences among vaccination intentions (*p* < 0.001).

### 3.2 Percentage of two or more vaccinations received according to pre-approval intention

**Figure 2:**
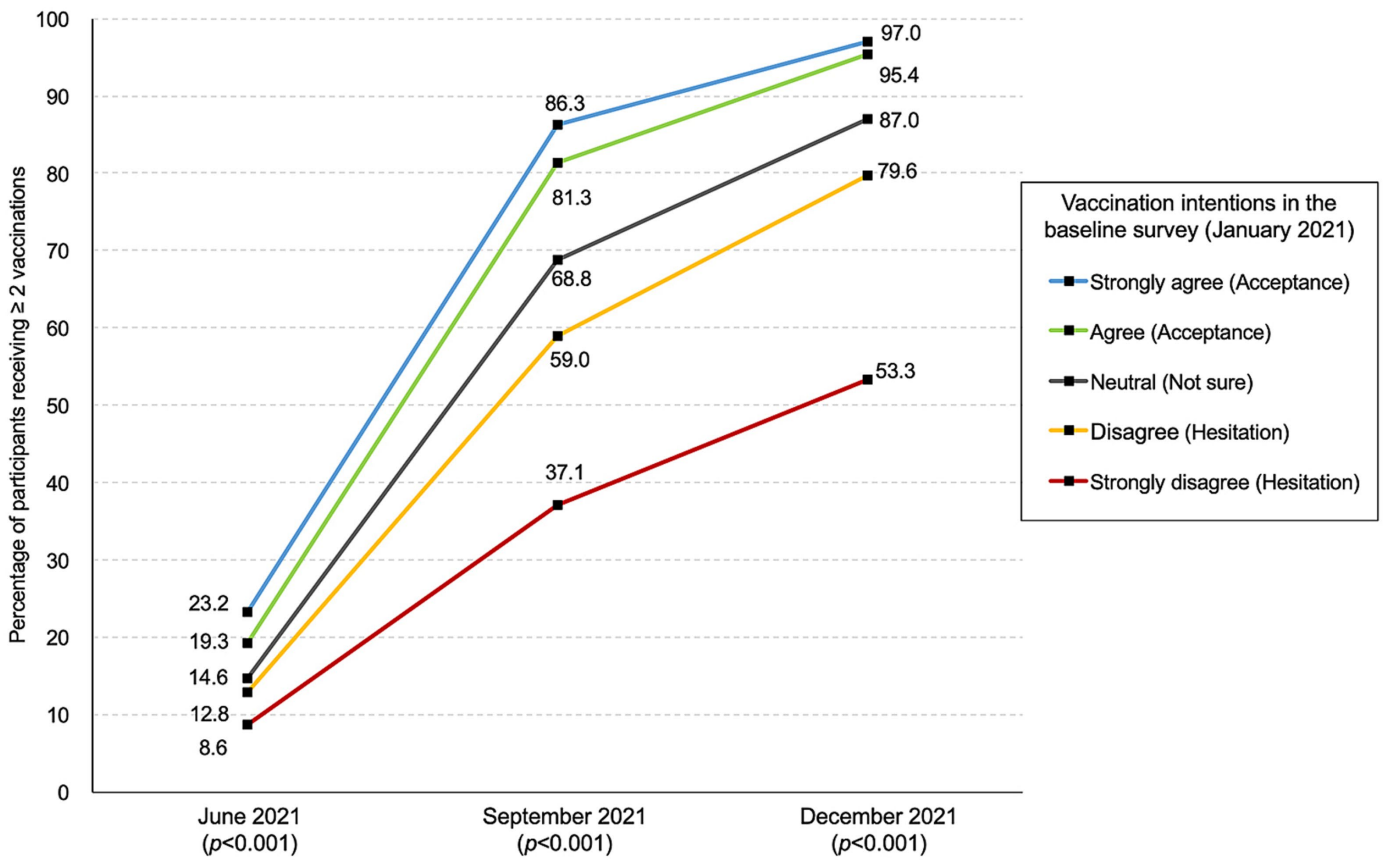
Percentage of participants receiving two or more vaccinations stratified by vaccination intention in January.

The changes in the percentage of subjects receiving ≥2 vaccinations by pre-approval intention are summarized in Figure 2. A significant difference was observed after June 2021 (*p* < 0.001). As of December 2021, 95.8% (593/619), 87.0% (1,177/1,353), and 72.2% (1,370/1,898) of those responding with “acceptance,” “not sure,” and “hesitation” had been vaccinated, respectively (*p* < 0.001) (see Figure 2).

### 3.3 Significant factors that predicted the completion of ≥2 COVID-19 vaccinations

Table 3 highlights significant predictors for ≥2 vaccine doses by December 2021, based on multivariate logistic regression analysis. Overall, significant factors associated with COVID-19 vaccine uptake included “social norms” (AOR, 1.80; 95% CI, 1.56–2.08; *p* < 0.001), “confidence” (AOR, 1.39; 95% CI, 1.18–1.64; *p* < 0.001), and “structural constraints” (AOR, 0.80; 95% CI, 0.70–0.91; *p* = 0.001).

**Table 3:** Significant factors that predicted the completion of more than two doses of COVID-19 vaccine within 1 year after its introduction (booster approved December 16, 2021).

	AOR^a^	95% CI	*p* value^b^
Overall (*n* = 3,870)			
Social norms	1.80	1.56–2.08	<0.001
Confidence	1.39	1.18–1.64	<0.001
Structural constraints	0.80	0.70–0.91	0.001
Acceptance^c^ (*n* = 1,898)			
Fever or swelling	1.82	1.28–2.60	0.001
Importance	1.60	1.12–2.30	0.011
Adverse reactions	0.65	0.46–0.91	0.011
Not sure^d^ (*n* = 1,353)			
Social norms	1.70	1.26–2.31	0.001
Handwashing	0.75	0.61–0.91	0.004
Structural constraints	0.67	0.55–0.83	<0.001
Hesitation^e^ (*n* = 619)			
Social norms	2.43	1.83–3.22	<0.001
Confidence	1.44	1.10–1.88	0.008
Knowledge	0.69	0.53–0.88	0.003

AOR, adjusted odds ratio; CI, confidence interval.

a Adjusted for gender, age, marital status, children, household income category, and presence of diseases under treatment using multivariable logistic regression analysis with a backward stepwise algorithm.

b Significance level is defined as 0.013 using the Bonferroni method.

c Acceptance includes the pre-approval intentions “strongly agree” and “agree”.

d Not sure is the pre-approval intention “neutral”.

e Hesitation includes the pre-approval intentions “strongly disagree” and “disagree”.

In the group of individuals classified as “acceptance,” significant factors associated with COVID-19 vaccination included “fever or swelling” (AOR, 1.82; 95% CI, 1.28–2.60; *p* = 0.001), “importance” (AOR, 1.60; 95% CI, 1.12–2.30; *p* = 0.011), and “adverse reactions” (AOR, 0.65; 95% CI, 0.46–0.91; *p* = 0.011). In the group classified as “not sure,” significant factors associated with COVID-19 vaccination included “social norms” (AOR, 1.70; 95% CI, 1.26–2.31; *p* = 0.001), “handwashing” (AOR, 0.75; 95% CI, 0.61–0.91; *p* = 0.004), and “structural constraints” (AOR, 0.67; 95% CI, 0.55–0.83; *p* < 0.001). In the group classified as showing “hesitation,” significant factors associated with COVID-19 vaccination included “social norms” (AOR, 2.43; 95% CI, 1.83–3.22; *p* < 0.001), “confidence” (AOR, 1.44; 95% CI, 1.10–1.88; *p* = 0.008), and “knowledge” (AOR, 0.69; 95% CI, 0.53–0.88; *p* = 0.003).

## 4 Discussion

Similar studies from the US and Africa (29, 30), both of which were short-term (approximately two months), reported changes in population proportions. To account for their findings, we first investigated whether people who were “hesitant” before the vaccine was approved started getting vaccinated within a year. Our study involved a one-year longitudinal Web-based observational study in Japan which tracked vaccination status based on pre- and post-approval vaccination intention (January 2021, June 2021, September 2021, and December 2021) and identified factors predicting the receipt of ≥2 vaccine doses, especially in those initially hesitant. Approximately 96, 87, and 72% of those who reported “acceptance,” “not sure,” or “hesitation” before vaccination had been vaccinated after 1 year, respectively. Overall, completing ≥2 vaccine doses was associated with “social norms” and “confidence,” whereas not completing ≥2 vaccine doses was associated with “structural constraints.” In the “hesitation” group, completing ≥2 vaccine doses was associated with “social norms” and “confidence,” whereas not completing ≥2 vaccine doses was associated with “knowledge”.

We found that irrespective of vaccination intention before the introduction of the vaccine, the majority had completed two doses within one year after the introduction of the vaccine. Specifically, 53.3% of those who answered “strongly disagree” were vaccinated. Similar to our findings, vaccine hesitancy decreased with the onset of COVID-19 vaccination according to the U.S. national survey conducted from October 2020 to March 2021 (31) and the CHASING COVID Cohort study (32). Therefore, it has been shown that hesitance is not the same as refusal, as stated by Larson et al. (33). As we previously reported (20), vaccine hesitancy may decrease during the deployment phase.

In the stratified groups of individuals classified as “hesitant” and “not sure,” the common factor that may drive vaccination (i.e., an AOR > 1 with a statistically significant *p*-value) was “social norms.” Similar to our findings, a U.S. study with 444 participants (34) also found that peer influence significantly affected vaccination decisions. Furthermore, in a study by Nomura et al. (35), individuals who initially had no intention of getting vaccinated or were unsure about vaccination (*n* = 8,077) were later influenced by the vaccination status of those close to them.

In the group of individuals classified as showing “hesitation,” the other factor of vaccination intention before approval was “confidence.” Furthermore, in the group classified as showing “acceptance,” the predictors of vaccination intention before approval included “fever or swelling.” Rane and colleagues (32) discovered that the percentage of vaccine delays decreased as more information on vaccine safety and effectiveness became available, and as more people received vaccines without issues. Hence, we conclude that these factors, which predict receipt of both doses of the vaccine, are associated with individuals observing the vaccination experiences and safety (even with minor adverse effects) of those around them.

In the group of individuals classified as showing “acceptance,” predictive factors included “importance.” Similarly, previous studies have reported that an awareness of the risks associated with the illness and the severity of its consequences may have resulted in behavioral change (36–38).

In the group of individuals classified as “not sure,” factors that may have hindered vaccination (i.e., an AOR < 1 with a statistically significant *p*-value) were observed for “structural constraints.” When considering predictors of vaccination, a 2010 meta-analysis of The Health Belief Model (HBM) included “barriers” as factors influencing vaccination (38). Additionally, a systematic review conducted during previous pandemics indicated that factors affecting vaccination included age and ease of vaccination (39). Three cross-sectional studies conducted by Betsch et al. in Germany and the U.S. (40) found that “barriers” (both structural and psychological obstacles), such as costs, travel time, inconvenience, and other time constraints deterred individuals from getting vaccinated (40). These findings suggest that significant “barriers” play an important role in preventing vaccination. In Japan, vaccinations are provided free of charge to citizens and foreigners with residence status (41). Eligible individuals receive vaccination vouchers from the government through the mail, allowing them to schedule a convenient location and time for vaccination. This system may have effectively reduced vaccination barriers.

In the group of individuals classified as showing “acceptance,” factors that may have hindered vaccination were observed for “adverse reactions.” Likewise, concerns about potential side effects have been identified as a deterrent to vaccination in national surveys conducted in the United States (32), among Saudi Arabian citizens (42), and in a Scoping Review of the African continent (43). Although the side effects of mRNA vaccines are less severe than those of conventional vaccines, they often include fever and other side effects (5, 6, 9). Therefore, there is an urgent need to develop a vaccine with fewer side effects.

In the group of individuals classified as showing “hesitation,” having “knowledge” about COVID-19 was significantly associated with a lower likelihood of receiving ≥2 vaccine doses, but no such association was observed in the groups of individuals classified as “not sure” or “acceptance.” Similarly, a survey of American adults also found that having an accurate understanding of COVID-19 was associated with a higher likelihood of getting vaccinated (44). Therefore, the lack of accurate information appears to act as a barrier to vaccination. Moreover, trust in specific sources of COVID-19 information was identified as a strong predictor (45). This underscores the importance of providing reliable information about vaccine-targeted diseases to boost vaccination rates. However, people who avoided vaccination before the introduction of the vaccine and did not get vaccinated by December 2021 may not have received reliable information about COVID-19 due to incorrect information obtained from sources such as social media. In the future, it will also be necessary to pay attention to how information is acquired (13), and whether it is always accurate.

It is of the utmost importance to utilize these findings in the development of future strategies. Enhancing vaccination rates necessitates the widespread dissemination of COVID-19 knowledge, promotion of confidence in vaccines, and reduction in structural barriers which contribute to the formation of societal norms. As these social norms take shape, we can expect further improvements in vaccination uptake.

There are three strategies which can be employed to increase vaccination rates. The first is healthcare workers’ behavioral changes. Cross-sector and cross-role communication among healthcare workers, especially those of generation X, is likely to lead to the dissemination of accurate knowledge about COVID-19 vaccines and build trust in them (46, 47), ultimately resulting in increased vaccination rates. The second is to implement campaigns promoting simultaneous vaccination with the influenza vaccine (48). While evidence regarding simultaneous administration of influenza and COVID-19 vaccines was insufficient as of 2022 (48), a prospective observational study conducted in Italy in 2023 involving 942 healthcare workers in community hospitals demonstrated that simultaneous vaccination does not affect the safety or efficacy of COVID-19 vaccines (25). The third strategy is the enforcement of mandatory COVID-19 vaccination certification (showing proof of vaccination, recent negative test, or evidence of recovery) with restricted access to certain environments (49). Compared to campaigns and interventions targeting healthcare workers, mandatory vaccination has the potential to significantly increase vaccination rates even among vaccine-hesitant individuals, thereby serving as an additional policy tool to enhance herd immunity at the population level.

This study has several strengths: it spanned one year before vaccine approval, included four survey points during the COVID-19 pandemic, had participants from various regions across the country, and maintained a substantial sample size. On the other hand, it also had some limitations, which affect the generalization of our results. First, a degree of response bias may be present arising from the Web-based design of the study. For instance, subjects were limited to those who could use the Internet. Second, sampling bias may be present, given that the follow-up rate was 53.7%. Participants in the entire study were more likely to be male than those lost to follow-up (51.7% [1,999/3,870 individuals] vs. 41.8% [1,396/3,340 individuals], *p* < 0.001), and were older (36.5% [1,411/3,870 individuals] vs. 22.4% [747/3,340 individuals], *p* < 0.001). Furthermore, the survey was done online, potentially favoring those comfortable with the Internet. Nevertheless, the fact that the proportion of those who received ≥2 vaccinations closely matches government statistics (50) suggests that, if present, any selection bias is likely acceptable. Third, a degree of measurement bias may be present, due to the lack of monitoring of the Web-based data collection. Thus, it is not possible to check the response environment or attitude of participants, and this may have influenced the quality of the data. Despite these limitations, our findings provide valuable insights into vaccination behavior and public health strategy, particularly in the context of the COVID-19 pandemic in Japan.

In conclusion, approximately 96, 87, and 72% of the stratified group of individuals classified as showing “acceptance,” “not sure,” or “hesitation” before vaccination had been vaccinated after 1 year, respectively. Overall, receipt of ≥2 vaccine doses was associated with factors like social norms and confidence, whereas not receiving ≥2 vaccine doses was associated with structural constraints. For those who were initially hesitant about vaccination, COVID-19 knowledge, social norms, and vaccine safety confidence were influential factors that led to vaccination. Improving vaccination rates requires the widespread distribution of knowledge about COVID-19 and its safety and minimization of structural constraints. The rise in vaccination rates is associated with social norms, and we look forward to further enhancements in these rates. Our findings may help improve vaccine uptake in future pandemics.

## Data availability statement

The data presented in this study is available on request from the corresponding author (MH).

## Ethics statement

The studies involving humans were approved by the Ethics Committee of Saga University (approval number: R2–24; date of approval: 30 November 2020). The studies were conducted in accordance with the local legislation and institutional requirements. Written informed consent for participation in this study was provided by the participants’ legal guardians/next of kin.

## Author contributions

TK: Formal analysis, Investigation, Validation, Visualization, Writing – original draft. MT: Conceptualization, Data curation, Formal analysis, Investigation, Methodology, Project administration, Resources, Software, Validation, Visualization, Writing – original draft. AM: Supervision, Writing – review & editing. TN: Supervision, Writing – review & editing. YH: Supervision, Writing – review & editing. MH: Conceptualization, Data curation, Formal analysis, Funding acquisition, Investigation, Methodology, Project administration, Resources, Validation, Visualization, Writing – original draft.
