# Supplementary material for: Predictive factors of coronavirus disease (COVID-19) vaccination series completion: a one-year longitudinal web-based observational study in Japan
Source: Front Public Health. 2024 Feb 29;12:1348170. doi: 10.3389/fpubh.2024.1348170 (PMC10937344; doi:10.3389/fpubh.2024.1348170)
Supplement: Supplementary file 1 [file Table_1.DOCX]

Supplementary Material

**Supplemental Table 1.** Responses to factors stratified by intention to receive COVID-19 before vaccine approval (January).

|  |  | S.D. (*n*=197) | | | D (*n*=422) | | | N (*n*=1,353) | | | A (*n*=1,360) | | | S.A. (*n*=538) | | *p* value^a^ |
| --- | --- | --- | --- | --- | --- | --- | --- | --- | --- | --- | --- | --- | --- | --- | --- | --- |
| *Recognition of COVID-19 (8 items)* | | | | | | | | | | | | | | | | |
| Knowledge | S.D | 19 | (9.6%) | 12 | | (2.8%) | 50 | | (3.7%) | 33 | | (2.4%) | 29 | | (5.4%) | <0.001 |
|  | D | 45 | (22.8%) | 123 | | (29.1%) | 328 | | (24.2%) | 312 | | (22.9%) | 107 | | (19.9%) |  |
|  | N | 79 | (40.1%) | 187 | | (44.3%) | 665 | | (49.2%) | 648 | | (47.6%) | 204 | | (37.9%) |  |
|  | A | 42 | (21.3%) | 95 | | (22.5%) | 284 | | (21.0%) | 336 | | (24.7%) | 158 | | (29.4%) |  |
|  | S.A | 12 | (6.1%) | 5 | | (1.2%) | 26 | | (1.9%) | 31 | | (2.3%) | 40 | | (7.4%) |  |
| Symptoms | S.D | 59 | (29.9%) | 85 | | (20.1%) | 235 | | (17.4%) | 220 | | (16.2%) | 131 | | (24.3%) | <0.001 |
|  | D | 88 | (44.7%) | 248 | | (58.8%) | 729 | | (53.9%) | 765 | | (56.3%) | 258 | | (48.0%) |  |
|  | N | 34 | (17.3%) | 61 | | (14.5%) | 289 | | (21.4%) | 242 | | (17.8%) | 84 | | (15.6%) |  |
|  | A | 7 | (3.6%) | 22 | | (5.2%) | 76 | | (5.6%) | 106 | | (7.8%) | 40 | | (7.4%) |  |
|  | S.A | 9 | (4.6%) | 6 | | (1.4%) | 24 | | (1.8%) | 27 | | (2.0%) | 25 | | (4.6%) |  |
| Mild illness | S.D | 25 | (12.7%) | 34 | | (8.1%) | 88 | | (6.5%) | 116 | | (8.5%) | 99 | | (18.4%) | <0.001 |
|  | D | 44 | (22.3%) | 112 | | (26.5%) | 328 | | (24.2%) | 397 | | (29.2%) | 132 | | (24.5%) |  |
|  | N | 41 | (20.8%) | 133 | | (31.5%) | 484 | | (35.8%) | 372 | | (27.4%) | 128 | | (23.8%) |  |
|  | A | 56 | (28.4%) | 125 | | (29.6%) | 409 | | (30.2%) | 435 | | (32.0%) | 146 | | (27.1%) |  |
|  | S.A | 31 | (15.7%) | 18 | | (4.3%) | 44 | | (3.3%) | 40 | | (2.9%) | 33 | | (6.1%) |  |
| Severe illness | S.D | 7 | (3.6%) | 2 | | (0.5%) | 8 | | (0.6%) | 6 | | (0.4%) | 3 | | (0.6%) | <0.001 |
|  | D | 9 | (4.6%) | 21 | | (5.0%) | 27 | | (2.0%) | 25 | | (1.8%) | 9 | | (1.7%) |  |
|  | N | 21 | (10.7%) | 31 | | (7.3%) | 126 | | (9.3%) | 66 | | (4.9%) | 21 | | (3.9%) |  |
|  | A | 71 | (36.0%) | 214 | | (50.7%) | 653 | | (48.3%) | 713 | | (52.4%) | 181 | | (33.6%) |  |
|  | S.A | 89 | (45.2%) | 154 | | (36.5%) | 539 | | (39.8%) | 550 | | (40.4%) | 324 | | (60.2%) |  |
| Easily infectious | S.D | 5 | (2.5%) | 5 | | (1.2%) | 11 | | (0.8%) | 12 | | (0.9%) | 7 | | (1.3%) | <0.001 |
|  | D | 9 | (4.6%) | 16 | | (3.8%) | 23 | | (1.7%) | 30 | | (2.2%) | 6 | | (1.1%) |  |
|  | N | 27 | (13.7%) | 25 | | (5.9%) | 101 | | (7.5%) | 40 | | (2.9%) | 15 | | (2.8%) |  |
|  | A | 61 | (31.0%) | 203 | | (48.1%) | 624 | | (46.1%) | 689 | | (50.7%) | 143 | | (26.6%) |  |
|  | S.A | 95 | (48.2%) | 173 | | (41.0%) | 594 | | (43.9%) | 589 | | (43.3%) | 367 | | (68.2%) |  |
| Worried about getting | S.D | 24 | (12.2%) | 9 | | (2.1%) | 22 | | (1.6%) | 8 | | (0.6%) | 3 | | (0.6%) | <0.001 |
|  | D | 14 | (7.1%) | 37 | | (8.8%) | 57 | | (4.2%) | 37 | | (2.7%) | 16 | | (3.0%) |  |
|  | N | 36 | (18.3%) | 43 | | (10.2%) | 180 | | (13.3%) | 112 | | (8.2%) | 36 | | (6.7%) |  |
|  | A | 68 | (34.5%) | 222 | | (52.6%) | 678 | | (50.1%) | 760 | | (55.9%) | 203 | | (37.7%) |  |
|  | S.A | 55 | (27.9%) | 111 | | (26.3%) | 416 | | (30.7%) | 443 | | (32.6%) | 280 | | (52.0%) |  |
| May get | S.D | 16 | (8.1%) | 7 | | (1.7%) | 16 | | (1.2%) | 10 | | (0.7%) | 8 | | (1.5%) | <0.001 |
|  | D | 29 | (14.7%) | 37 | | (8.8%) | 79 | | (5.8%) | 88 | | (6.5%) | 28 | | (5.2%) |  |
|  | N | 52 | (26.4%) | 143 | | (33.9%) | 515 | | (38.1%) | 432 | | (31.8%) | 145 | | (27.0%) |  |
|  | A | 62 | (31.5%) | 195 | | (46.2%) | 611 | | (45.2%) | 723 | | (53.2%) | 240 | | (44.6%) |  |
|  | S.A | 38 | (19.3%) | 40 | | (9.5%) | 132 | | (9.8%) | 107 | | (7.9%) | 117 | | (21.7%) |  |
| Repeated infections | S.D | 96 | (48.7%) | 112 | | (26.5%) | 381 | | (28.2%) | 290 | | (21.3%) | 197 | | (36.6%) | <0.001 |
|  | D | 64 | (32.5%) | 232 | | (55.0%) | 685 | | (50.6%) | 771 | | (56.7%) | 232 | | (43.1%) |  |
|  | N | 28 | (14.2%) | 63 | | (14.9%) | 249 | | (18.4%) | 229 | | (16.8%) | 75 | | (13.9%) |  |
|  | A | 8 | (4.1%) | 14 | | (3.3%) | 32 | | (2.4%) | 63 | | (4.6%) | 28 | | (5.2%) |  |
|  | S.A | 1 | (0.5%) | 1 | | (0.2%) | 6 | | (0.4%) | 7 | | (0.5%) | 6 | | (1.1%) |  |
| *Awareness of the COVID-19 vaccine (7 items)* | | | | | | | | | | | | | | | | |
| Preventing severe illness | S.D | 34 | (17.3%) | 7 | | (1.7%) | 11 | | (0.8%) | 9 | | (0.7%) | 5 | | (0.1%) |  |
|  | D | 31 | (15.7%) | 53 | | (12.6%) | 72 | | (5.3%) | 67 | | (4.9%) | 15 | | (0.3%) |  |
|  | N | 55 | (27.9%) | 130 | | (30.8%) | 431 | | (31.9%) | 174 | | (12.8%) | 56 | | (1.0%) |  |
|  | A | 64 | (32.5%) | 208 | | (49.3%) | 765 | | (56.5%) | 986 | | (72.5%) | 272 | | (5.0%) |  |
|  | S.A | 13 | (6.6%) | 24 | | (5.7%) | 74 | | (5.5%) | 124 | | (9.1%) | 190 | | (3.5%) |  |
| Prevention of infection | S.D | 46 | (23.4%) | 16 | | (3.8%) | 37 | | (2.7%) | 21 | | (1.5%) | 12 | | (2.2%) | <0.001 |
|  | D | 45 | (22.8%) | 106 | | (25.1%) | 174 | | (12.9%) | 134 | | (9.9%) | 39 | | (7.2%) |  |
|  | N | 52 | (26.4%) | 145 | | (34.4%) | 512 | | (37.8%) | 228 | | (16.8%) | 60 | | (11.2%) |  |
|  | A | 48 | (24.4%) | 147 | | (34.8%) | 594 | | (43.9%) | 926 | | (68.1%) | 292 | | (54.3%) |  |
|  | S.A | 6 | (3.0%) | 8 | | (1.9%) | 36 | | (2.7%) | 51 | | (3.8%) | 135 | | (25.1%) |  |
| Preventing relatives’ infection | S.D | 42 | (21.3%) | 35 | | (8.3%) | 73 | | (5.4%) | 63 | | (4.6%) | 38 | | (7.1%) | <0.001 |
|  | D | 50 | (25.4%) | 113 | | (26.8%) | 235 | | (17.4%) | 227 | | (16.7%) | 60 | | (7.1%) |  |
|  | N | 63 | (32.0%) | 174 | | (41.2%) | 543 | | (40.1%) | 239 | | (17.6%) | 70 | | (7.1%) |  |
|  | A | 37 | (18.8%) | 99 | | (23.5%) | 479 | | (35.4%) | 774 | | (56.9%) | 214 | | (7.1%) |  |
|  | S.A | 5 | (2.5%) | 1 | | (0.2%) | 23 | | (1.7%) | 57 | | (4.2%) | 156 | | (7.1%) |  |
| Prevention of spread | S.D | 38 | (19.3%) | 12 | | (2.8%) | 22 | | (1.6%) | 16 | | (1.2%) | 9 | | (1.7%) | <0.001 |
|  | D | 40 | (20.3%) | 88 | | (20.9%) | 146 | | (10.8%) | 95 | | (7.0%) | 31 | | (5.8%) |  |
|  | N | 78 | (39.6%) | 173 | | (41.0%) | 552 | | (40.8%) | 242 | | (17.8%) | 65 | | (12.1%) |  |
|  | A | 37 | (18.8%) | 144 | | (34.1%) | 604 | | (44.6%) | 932 | | (68.5%) | 265 | | (49.3%) |  |
|  | S.A | 4 | (2.0%) | 5 | | (1.2%) | 29 | | (2.1%) | 75 | | (5.5%) | 168 | | (31.2%) |  |
| Adverse reactions | S.D | 3 | (1.5%) | 1 | | (0.2%) | 3 | | (0.2%) | 7 | | (0.5%) | 18 | | (3.3%) | <0.001 |
|  | D | 2 | (1.0%) | 21 | | (5.0%) | 33 | | (2.4%) | 84 | | (6.2%) | 79 | | (14.7%) |  |
|  | N | 6 | (3.0%) | 30 | | (7.1%) | 193 | | (14.3%) | 281 | | (20.7%) | 126 | | (23.4%) |  |
|  | A | 30 | (15.2%) | 166 | | (39.3%) | 661 | | (48.9%) | 742 | | (54.6%) | 217 | | (40.3%) |  |
|  | S.A | 156 | (79.2%) | 204 | | (48.3%) | 463 | | (34.2%) | 246 | | (18.1%) | 98 | | (18.2%) |  |
| Fever or swelling | S.D | 5 | (2.5%) | 5 | | (1.2%) | 6 | | (0.4%) | 7 | | (0.5%) | 7 | | (1.3%) | <0.001 |
|  | D | 5 | (2.5%) | 39 | | (9.2%) | 69 | | (5.1%) | 113 | | (8.3%) | 62 | | (11.5%) |  |
|  | N | 58 | (29.4%) | 160 | | (37.9%) | 685 | | (50.6%) | 675 | | (49.6%) | 228 | | (42.4%) |  |
|  | A | 62 | (31.5%) | 179 | | (42.4%) | 518 | | (38.3%) | 503 | | (37.0%) | 190 | | (35.3%) |  |
|  | S.A | 67 | (34.0%) | 39 | | (9.2%) | 75 | | (5.5%) | 62 | | (4.6%) | 51 | | (9.5%) |  |
| Social norm | S.D | 123 | (62.4%) | 13 | | (3.1%) | 7 | | (0.5%) | 2 | | (0.1%) | 2 | | (0.4%) | <0.001 |
|  | D | 46 | (23.4%) | 200 | | (47.4%) | 59 | | (4.4%) | 15 | | (1.1%) | 9 | | (1.7%) |  |
|  | N | 16 | (8.1%) | 154 | | (36.5%) | 759 | | (56.1%) | 98 | | (7.2%) | 34 | | (6.3%) |  |
|  | A | 8 | (4.1%) | 53 | | (12.6%) | 498 | | (36.8%) | 1045 | | (76.8%) | 120 | | (22.3%) |  |
|  | S.A | 4 | (2.0%) | 2 | | (0.5%) | 30 | | (2.2%) | 200 | | (14.7%) | 373 | | (69.3%) |  |
| *Perceptions for COVID-19-related health preventive behavior (8 items)* | | | | | | | | | | | | | | | | |
| Social distance | S.D | 9 | (4.6%) | 1 | | (0.2%) | 7 | | (0.5%) | 4 | | (0.3%) | 3 | | (0.6%) | <0.001 |
|  | D | 12 | (6.1%) | 17 | | (4.0%) | 27 | | (2.0%) | 21 | | (1.5%) | 10 | | (1.9%) |  |
|  | N | 25 | (12.7%) | 41 | | (9.7%) | 200 | | (14.8%) | 118 | | (8.7%) | 34 | | (6.3%) |  |
|  | A | 66 | (33.5%) | 243 | | (57.6%) | 782 | | (57.8%) | 871 | | (64.0%) | 263 | | (48.9%) |  |
|  | S.A | 85 | (43.1%) | 120 | | (28.4%) | 337 | | (24.9%) | 346 | | (25.4%) | 228 | | (42.4%) |  |
| Handwashing | S.D | 29 | (14.7%) | 17 | | (4.0%) | 44 | | (3.3%) | 26 | | (1.9%) | 17 | | (3.2%) | <0.001 |
|  | D | 28 | (14.2%) | 94 | | (22.3%) | 203 | | (15.0%) | 214 | | (15.7%) | 74 | | (13.8%) |  |
|  | N | 42 | (21.3%) | 88 | | (20.9%) | 440 | | (32.5%) | 390 | | (28.7%) | 129 | | (24.0%) |  |
|  | A | 61 | (31.0%) | 169 | | (40.0%) | 514 | | (38.0%) | 570 | | (41.9%) | 207 | | (38.5%) |  |
|  | S.A | 37 | (18.8%) | 54 | | (12.8%) | 152 | | (11.2%) | 160 | | (11.8%) | 111 | | (20.6%) |  |
| Hand sanitizer | S.D | 25 | (12.7%) | 8 | | (1.9%) | 17 | | (1.3%) | 9 | | (0.7%) | 4 | | (0.7%) | <0.001 |
|  | D | 10 | (5.1%) | 52 | | (12.3%) | 73 | | (5.4%) | 61 | | (4.5%) | 20 | | (3.7%) |  |
|  | N | 32 | (16.2%) | 54 | | (12.8%) | 196 | | (14.5%) | 136 | | (10.0%) | 41 | | (7.6%) |  |
|  | A | 78 | (39.6%) | 214 | | (50.7%) | 755 | | (55.8%) | 811 | | (59.6%) | 253 | | (47.0%) |  |
|  | S.A | 52 | (26.4%) | 94 | | (22.3%) | 312 | | (23.1%) | 343 | | (25.2%) | 220 | | (40.9%) |  |
| Wearing a mask | S.D | 10 | (5.1%) | 3 | | (0.7%) | 2 | | (0.1%) | 3 | | (0.2%) | 1 | | (0.2%) | <0.001 |
|  | D | 4 | (2.0%) | 7 | | (1.7%) | 12 | | (0.9%) | 11 | | (0.8%) | 1 | | (0.2%) |  |
|  | N | 12 | (6.1%) | 18 | | (4.3%) | 79 | | (5.8%) | 40 | | (2.9%) | 11 | | (2.0%) |  |
|  | A | 55 | (27.9%) | 151 | | (35.8%) | 532 | | (39.3%) | 562 | | (41.3%) | 120 | | (22.3%) |  |
|  | S.A | 116 | (58.9%) | 243 | | (57.6%) | 728 | | (53.8%) | 744 | | (54.7%) | 405 | | (75.3%) |  |
| Indoor ventilating | S.D | 23 | (11.7%) | 13 | | (3.1%) | 29 | | (2.1%) | 15 | | (1.1%) | 13 | | (2.4%) | <0.001 |
|  | D | 19 | (9.6%) | 64 | | (15.2%) | 147 | | (10.9%) | 130 | | (9.6%) | 52 | | (9.7%) |  |
|  | N | 39 | (19.8%) | 98 | | (23.2%) | 410 | | (30.3%) | 323 | | (23.8%) | 102 | | (19.0%) |  |
|  | A | 73 | (37.1%) | 178 | | (42.2%) | 590 | | (43.6%) | 707 | | (52.0%) | 233 | | (43.3%) |  |
|  | S.A | 43 | (21.8%) | 69 | | (16.4%) | 177 | | (13.1%) | 185 | | (13.6%) | 138 | | (25.7%) |  |
| Avoiding gatherings | S.D | 11 | (5.6%) | 4 | | (0.9%) | 7 | | (0.5%) | 9 | | (0.7%) | 2 | | (0.4%) | <0.001 |
|  | D | 3 | (1.5%) | 5 | | (1.2%) | 14 | | (1.0%) | 9 | | (0.7%) | 5 | | (0.9%) |  |
|  | N | 10 | (5.1%) | 27 | | (6.4%) | 89 | | (6.6%) | 52 | | (3.8%) | 16 | | (3.0%) |  |
|  | A | 33 | (16.8%) | 127 | | (30.1%) | 391 | | (28.9%) | 471 | | (34.6%) | 76 | | (14.1%) |  |
|  | S.A | 140 | (71.1%) | 259 | | (61.4%) | 852 | | (63.0%) | 819 | | (60.2%) | 439 | | (81.6%) |  |
| Going out | S.D | 7 | (3.6%) | 0 | | (0.0%) | 7 | | (0.5%) | 5 | | (0.4%) | 4 | | (0.7%) | <0.001 |
|  | D | 3 | (1.5%) | 6 | | (1.4%) | 23 | | (1.7%) | 13 | | (1.0%) | 8 | | (1.5%) |  |
|  | N | 32 | (16.2%) | 70 | | (16.6%) | 195 | | (14.4%) | 126 | | (9.3%) | 44 | | (8.2%) |  |
|  | A | 58 | (29.4%) | 169 | | (40.0%) | 599 | | (44.3%) | 697 | | (51.3%) | 154 | | (28.6%) |  |
|  | S.A | 97 | (49.2%) | 177 | | (41.9%) | 529 | | (39.1%) | 519 | | (38.2%) | 328 | | (61.0%) |  |
| Information | S.D | 13 | (6.6%) | 4 | | (0.9%) | 12 | | (0.9%) | 3 | | (0.2%) | 3 | | (0.6%) | <0.001 |
|  | D | 13 | (6.6%) | 24 | | (5.7%) | 48 | | (3.5%) | 41 | | (3.0%) | 17 | | (3.2%) |  |
|  | N | 35 | (17.8%) | 98 | | (23.2%) | 324 | | (23.9%) | 225 | | (16.5%) | 61 | | (11.3%) |  |
|  | A | 72 | (36.5%) | 192 | | (45.5%) | 658 | | (48.6%) | 773 | | (56.8%) | 227 | | (42.2%) |  |
|  | S.A | 64 | (32.5%) | 104 | | (24.6%) | 311 | | (23.0%) | 318 | | (23.4%) | 230 | | (42.8%) |  |
| *Perceptions for COVID-19 vaccine reliability (14 items)* | | | | | | | | | | | | | | | | |
| Importance | S.D | 54 | (27.4%) | 9 | | (2.1%) | 6 | | (0.4%) | 8 | | (0.6%) | 1 | | (0.2%) | <0.001 |
|  | D | 39 | (19.8%) | 79 | | (18.7%) | 60 | | (4.4%) | 33 | | (2.4%) | 4 | | (0.7%) |  |
|  | N | 70 | (35.5%) | 215 | | (50.9%) | 726 | | (53.7%) | 239 | | (17.6%) | 42 | | (7.8%) |  |
|  | A | 24 | (12.2%) | 109 | | (25.8%) | 527 | | (39.0%) | 967 | | (71.1%) | 267 | | (49.6%) |  |
|  | S.A | 10 | (5.1%) | 10 | | (2.4%) | 34 | | (2.5%) | 113 | | (8.3%) | 224 | | (41.6%) |  |
| Effectiveness | S.D | 34 | (17.3%) | 1 | | (0.2%) | 8 | | (0.6%) | 4 | | (0.3%) | 1 | | (0.2%) | <0.001 |
|  | D | 39 | (19.8%) | 48 | | (11.4%) | 52 | | (3.8%) | 29 | | (2.1%) | 4 | | (0.7%) |  |
|  | N | 82 | (41.6%) | 201 | | (47.6%) | 612 | | (45.2%) | 203 | | (14.9%) | 41 | | (7.6%) |  |
|  | A | 36 | (18.3%) | 160 | | (37.9%) | 646 | | (47.7%) | 1035 | | (76.1%) | 312 | | (58.0%) |  |
|  | S.A | 6 | (3.0%) | 12 | | (2.8%) | 35 | | (2.6%) | 89 | | (6.5%) | 180 | | (33.5%) |  |
| Herd immunity | S.D | 83 | (42.1%) | 43 | | (10.2%) | 48 | | (3.5%) | 33 | | (2.4%) | 13 | | (2.4%) | <0.001 |
|  | D | 59 | (29.9%) | 181 | | (42.9%) | 358 | | (26.5%) | 181 | | (13.3%) | 42 | | (7.8%) |  |
|  | N | 47 | (23.9%) | 170 | | (40.3%) | 811 | | (59.9%) | 733 | | (53.9%) | 201 | | (37.4%) |  |
|  | A | 6 | (3.0%) | 23 | | (5.5%) | 134 | | (9.9%) | 398 | | (29.3%) | 233 | | (43.3%) |  |
|  | S.A | 2 | (1.0%) | 5 | | (1.2%) | 2 | | (0.1%) | 15 | | (1.1%) | 49 | | (9.1%) |  |
| Risk | S.D | 24 | (12.2%) | 7 | | (1.7%) | 19 | | (1.4%) | 17 | | (1.3%) | 9 | | (1.7%) | <0.001 |
|  | D | 26 | (13.2%) | 58 | | (13.7%) | 117 | | (8.6%) | 88 | | (6.5%) | 30 | | (5.6%) |  |
|  | N | 81 | (41.1%) | 152 | | (36.0%) | 490 | | (36.2%) | 257 | | (18.9%) | 69 | | (12.8%) |  |
|  | A | 53 | (26.9%) | 188 | | (44.5%) | 672 | | (49.7%) | 899 | | (66.1%) | 250 | | (46.5%) |  |
|  | S.A | 13 | (6.6%) | 17 | | (4.0%) | 55 | | (4.1%) | 99 | | (7.3%) | 180 | | (33.5%) |  |
| Anxiety | S.D | 5 | (2.5%) | 3 | | (0.7%) | 9 | | (0.7%) | 7 | | (0.5%) | 15 | | (2.8%) | <0.001 |
|  | D | 7 | (3.6%) | 16 | | (3.8%) | 49 | | (3.6%) | 130 | | (9.6%) | 72 | | (13.4%) |  |
|  | N | 25 | (12.7%) | 97 | | (23.0%) | 507 | | (37.5%) | 514 | | (37.8%) | 184 | | (34.2%) |  |
|  | A | 61 | (31.0%) | 229 | | (54.3%) | 662 | | (48.9%) | 632 | | (46.5%) | 226 | | (42.0%) |  |
|  | S.A | 99 | (50.3%) | 77 | | (18.2%) | 126 | | (9.3%) | 77 | | (5.7%) | 41 | | (7.6%) |  |
| Trust | S.D | 5 | (2.5%) | 1 | | (0.2%) | 7 | | (0.5%) | 14 | | (1.0%) | 26 | | (4.8%) | <0.001 |
|  | D | 7 | (3.6%) | 25 | | (5.9%) | 67 | | (5.0%) | 178 | | (13.1%) | 100 | | (18.6%) |  |
|  | N | 39 | (19.8%) | 151 | | (35.8%) | 673 | | (49.7%) | 636 | | (46.8%) | 237 | | (44.1%) |  |
|  | A | 47 | (23.9%) | 184 | | (43.6%) | 505 | | (37.3%) | 477 | | (35.1%) | 144 | | (26.8%) |  |
|  | S.A | 99 | (50.3%) | 61 | | (14.5%) | 101 | | (7.5%) | 55 | | (4.0%) | 31 | | (5.8%) |  |
| Confidence | S.D | 3 | (1.5%) | 1 | | (0.2%) | 6 | | (0.4%) | 14 | | (1.0%) | 19 | | (3.5%) | <0.001 |
|  | D | 1 | (0.5%) | 20 | | (4.7%) | 59 | | (4.4%) | 139 | | (10.2%) | 103 | | (19.1%) |  |
|  | N | 15 | (7.6%) | 67 | | (15.9%) | 289 | | (21.4%) | 378 | | (27.8%) | 142 | | (26.4%) |  |
|  | A | 41 | (20.8%) | 195 | | (46.2%) | 691 | | (51.1%) | 674 | | (49.6%) | 211 | | (39.2%) |  |
|  | S.A | 137 | (69.5%) | 139 | | (32.9%) | 308 | | (22.8%) | 155 | | (11.4%) | 63 | | (11.7%) |  |
| Distrust | S.D | 21 | (10.7%) | 37 | | (8.8%) | 96 | | (7.1%) | 208 | | (15.3%) | 220 | | (40.9%) | <0.001 |
|  | D | 32 | (16.2%) | 105 | | (24.9%) | 450 | | (33.3%) | 634 | | (46.6%) | 170 | | (31.6%) |  |
|  | N | 61 | (31.0%) | 162 | | (38.4%) | 503 | | (37.2%) | 317 | | (23.3%) | 76 | | (14.1%) |  |
|  | A | 41 | (20.8%) | 90 | | (21.3%) | 272 | | (20.1%) | 188 | | (13.8%) | 55 | | (10.2%) |  |
|  | S.A | 42 | (21.3%) | 28 | | (6.6%) | 32 | | (2.4%) | 13 | | (1.0%) | 17 | | (3.2%) |  |
| Structural constraints | S.D | 26 | (13.2%) | 27 | | (6.4%) | 114 | | (8.4%) | 151 | | (11.1%) | 151 | | (28.1%) | <0.001 |
|  | D | 42 | (21.3%) | 169 | | (40.0%) | 456 | | (33.7%) | 609 | | (44.8%) | 188 | | (34.9%) |  |
|  | N | 76 | (38.6%) | 163 | | (38.6%) | 624 | | (46.1%) | 454 | | (33.4%) | 141 | | (26.2%) |  |
|  | A | 31 | (15.7%) | 59 | | (14.0%) | 152 | | (11.2%) | 136 | | (10.0%) | 46 | | (8.6%) |  |
|  | S.A | 22 | (11.2%) | 4 | | (0.9%) | 7 | | (0.5%) | 10 | | (0.7%) | 12 | | (2.2%) |  |
| Psychological constraints | S.D | 4 | (2.0%) | 8 | | (1.9%) | 32 | | (2.4%) | 75 | | (5.5%) | 138 | | (25.7%) | <0.001 |
|  | D | 14 | (7.1%) | 62 | | (14.7%) | 258 | | (19.1%) | 569 | | (41.8%) | 225 | | (41.8%) |  |
|  | N | 50 | (25.4%) | 182 | | (43.1%) | 792 | | (58.5%) | 549 | | (40.4%) | 128 | | (23.8%) |  |
|  | A | 66 | (33.5%) | 154 | | (36.5%) | 244 | | (18.0%) | 150 | | (11.0%) | 35 | | (6.5%) |  |
|  | S.A | 63 | (32.0%) | 16 | | (3.8%) | 27 | | (2.0%) | 17 | | (1.3%) | 12 | | (2.2%) |  |
| Compliance | S.D | 22 | (11.2%) | 28 | | (6.6%) | 142 | | (10.5%) | 287 | | (21.1%) | 286 | | (53.2%) | <0.001 |
|  | D | 37 | (18.8%) | 162 | | (38.4%) | 621 | | (45.9%) | 815 | | (59.9%) | 177 | | (32.9%) |  |
|  | N | 85 | (43.1%) | 169 | | (40.0%) | 498 | | (36.8%) | 181 | | (13.3%) | 36 | | (6.7%) |  |
|  | A | 25 | (12.7%) | 52 | | (12.3%) | 82 | | (6.1%) | 60 | | (4.4%) | 26 | | (4.8%) |  |
|  | S.A | 28 | (14.2%) | 11 | | (2.6%) | 10 | | (0.7%) | 17 | | (1.3%) | 13 | | (2.4%) |  |
| Literacy | S.D | 41 | (20.8%) | 24 | | (5.7%) | 63 | | (4.7%) | 26 | | (1.9%) | 16 | | (3.0%) | <0.001 |
|  | D | 55 | (27.9%) | 165 | | (39.1%) | 352 | | (26.0%) | 315 | | (23.2%) | 103 | | (19.1%) |  |
|  | N | 69 | (35.0%) | 174 | | (41.2%) | 719 | | (53.1%) | 631 | | (46.4%) | 191 | | (35.5%) |  |
|  | A | 25 | (12.7%) | 53 | | (12.6%) | 207 | | (15.3%) | 368 | | (27.1%) | 190 | | (35.3%) |  |
|  | S.A | 7 | (3.6%) | 6 | | (1.4%) | 12 | | (0.9%) | 20 | | (1.5%) | 38 | | (7.1%) |  |
| Understanding necessity | S.D | 31 | (15.7%) | 6 | | (1.4%) | 22 | | (1.6%) | 10 | | (0.7%) | 8 | | (1.5%) | <0.001 |
|  | D | 29 | (14.7%) | 99 | | (23.5%) | 163 | | (12.0%) | 142 | | (10.4%) | 35 | | (6.5%) |  |
|  | N | 72 | (36.5%) | 150 | | (35.5%) | 635 | | (46.9%) | 411 | | (30.2%) | 114 | | (21.2%) |  |
|  | A | 54 | (27.4%) | 151 | | (35.8%) | 491 | | (36.3%) | 717 | | (52.7%) | 267 | | (49.6%) |  |
|  | S.A | 11 | (5.6%) | 16 | | (3.8%) | 42 | | (3.1%) | 80 | | (5.9%) | 114 | | (21.2%) |  |
| Understanding vaccinations | S.D | 29 | (14.7%) | 17 | | (4.0%) | 62 | | (4.6%) | 33 | | (2.4%) | 22 | | (4.1%) | <0.001 |
|  | D | 37 | (18.8%) | 143 | | (33.9%) | 315 | | (23.3%) | 264 | | (19.4%) | 77 | | (14.3%) |  |
|  | N | 73 | (37.1%) | 134 | | (31.8%) | 589 | | (43.5%) | 514 | | (37.8%) | 156 | | (29.0%) |  |
|  | A | 40 | (20.3%) | 117 | | (27.7%) | 360 | | (26.6%) | 507 | | (37.3%) | 222 | | (41.3%) |  |
|  | S.A | 18 | (9.1%) | 11 | | (2.6%) | 27 | | (2.0%) | 42 | | (3.1%) | 61 | | (11.3%) |  |

*S.D*, strongly disagree; *D*, disagree; *N*, not sure; *A*, agree; *S.A*, strongly agree; *COVID-19*, coronavirus disease.

^a^The significance level was defined as 0.001 using the Bonferroni method.

**
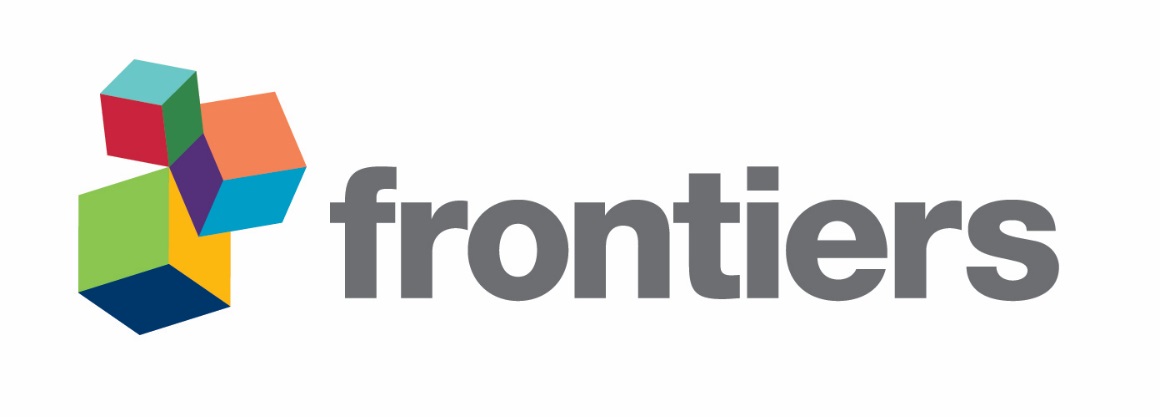
**
